# Lung and fissure shape is associated with age in healthy never-smoking adults aged 20–90 years

**DOI:** 10.1038/s41598-020-73117-w

**Published:** 2020-09-30

**Authors:** Mahyar Osanlouy, Alys R. Clark, Haribalan Kumar, Clair King, Margaret L. Wilsher, David G. Milne, Ken Whyte, Eric A. Hoffman, Merryn H. Tawhai

**Affiliations:** 1 Auckland Bioengineering Institute, University of Auckland, Auckland, 1010 New Zealand; 2 Respiratory Services, Auckland City Hospital, Auckland, 1010 New Zealand; 3 Faculty of Medical and Health Sciences, University of Auckland, Auckland, 1010 New Zealand; 4 Department of Radiology, Auckland City Hospital, Auckland, 1010 New Zealand; 5 Departments of Radiology and Bioengineering, University of Iowa, Iowa, 52242 USA

**Keywords:** Computational models, Predictive markers, Respiration, Biomedical engineering, Ageing

## Abstract

Lung shape could hold prognostic information for age-related diseases that affect lung tissue mechanics. We sought to quantify mean lung shape, its modes of variation, and shape associations with lung size, age, sex, and Body Mass Index (BMI) in healthy subjects across a seven-decade age span. Volumetric computed tomography from 83 subjects (49 M/34 F, BMI $24.7 \pm 2.7$) was used to derive two statistical shape models using a principal component analysis. One model included, and the other controlled for, lung volume. Volume made the strongest contribution to shape when it was included. Shape had a strong relationship with age but not sex when volume was controlled for, and BMI had only a small but significant association with shape. The first principal shape mode was associated with decrease in the antero-posterior dimension from base to apex. In older subjects this was rapid and obvious, whereas younger subjects had relatively more constant dimension. A shift of the fissures of both lungs in the basal direction was apparent for the older subjects, consistent with a change in tissue elasticity with age. This study suggests a quantifiable structure-function relationship for the healthy adult lung that can potentially be exploited as a normative description against which abnormal can be compared.

## Introduction

Advancing age is associated with increasing chest wall stiffness and changes to thorax shape due to calcification of costal cartilages, narrowing of intervertebral spaces, and increased dorsal kyphosis/anteroposterior diameter (‘barrel chest’)^1^. Given the tight apposition of the lung and chest wall, it is reasonable to expect that the lung changes shape along with the thorax. However, to date adult age-related lung shape has only been directly examined quantitatively using clinical 2D chest X-ray^2^ which cannot give a complete shape description.

Quantitative descriptions of normal lung shape, inter-subject variability, and age-related differences are important for several reasons. First, diseases with age-related prevalence that affect the lung tissue can develop with a regional preference; for example, preferentially apical for emphysema as a component of chronic obstructive pulmonary disease (COPD), or subpleural basal for idiopathic pulmonary fibrosis (IPF). In IPF this has been proposed to be related to locally high shear stress^3^, which itself depends upon the degree of lung inflation and change in shape associated with posture and chest wall expansion during breathing. Detailed testing of this hypothesis has been limited by a lack of description of the normal and pathological lung shape. Second, mechanical changes to the lung tissue and chest wall that accompany COPD or IPF affect the functional deformation of the lung tissue during breathing or breath-hold^4^. Lung and lobe shape—as well as the spatial distribution of abnormal tissue—could therefore hold prognostic information for staging or stratification of patients with these conditions. Finally, information on shape would improve automated image processing methods for detection of the pulmonary fissures, where fissure integrity, for example, is an important predictor of outcome for endobronchial valve treatment of severe emphysema^5^.

Despite its importance, descriptions of normal lung shape remain largely qualitative except for a relatively small number of studies (e.g.^6–8^). Several studies have suggested sexual dimorphism in ribcage morphology^7,9,10^ and to a small extent ($~7\%$)—in static supine lung shape^11^. Sex-related shape differences could be important for understanding the greater susceptibility of women to COPD^12^. Other studies of subjects with and without COPD have observed a dependence of diaphragm shape on age (16) and Body Mass Index (BMI)^13,14^ and an association between percent emphysema and cross-sectional area of the lung^12^. However, no study has yet considered the impact of healthy adult age on lung—rather than its ‘container’—shape.

Biological shape variations are often complex and highly challenging to interpret. Principal component analysis (PCA) is a mathematical method that has previously been used to describe the shape of the lung^8^ and other organs^15–17^. PCA simplifies the complex patterns of shape variation into independent variables (principal components), providing more-readily interpretable metrics of shape than simple linear dimensions. Here, we sought to quantify the average lung shape using a PCA to derive a statistical shape average model and its principal modes of shape variation for a cohort of healthy adults aged 20–93 years of age, and to determine whether lung shape in these subjects is associated with age, sex, or BMI.

## Methods

### Data acquisition and preprocessing

Imaging and pulmonary function testing data from 83 (34 M/49 F) never-smoking subjects aged 20–93 years, with no history of lung disease or injury to the lung or chest wall were analyzed. Data were retrospectively obtained from two studies with consistent protocols. 47 (19 M/28 F) subjects aged 50–93 years ($71.2 \pm 10.9$ years) were recruited with approval from the Northern A Health and Disability Ethics Committee, and data were acquired at Auckland City Hospital (New Zealand). A further 36 (15/21 M/F) subjects aged 20–49 years (mean $32.0 \pm 12$ years) were selected retrospectively from the University of Iowa Comprehensive Lung Imaging Center ‘Human Lung Atlas’ database (data acquired under NIH R01-HL-064368, E. A. Hoffman PI, following approval by the University of Iowa Institutional Review Board and Radiation Safety Committees). All the data acquisition and analysis methods were carried out in accordance with relevant guidelines and regulations. An informed consent was obtained from all subjects. Subject data were selected to provide as uniform as possible spread of age by decade and sex, within the constraints of the available data. Data comprised volumetric multi-detector row computed tomography (MDCT) at full inspiration, spirometry, and lung volumes by plethysmography. Exclusion criteria were $\hbox {BMI} > \, 30 \, {\hbox {kg/m}}^{2}$, a history of respiratory or cardiac disease, ever-smoking, $\hbox {FEV1} < 80\%$ predicted and FEV1/FVC ${<}$ lower limit of normal^18^. Anthropometric data are given in Table 1.

**Table 1:** Summary anthropometric data for a cohort of never-smoking subjects aged 20–93 years, used for lung shape analysis.

	Anthropometric data (mean ± SD, N = 83)
Age (years)	$53 \pm 22$
Sex (M/F)	$34\, (41\%) / 49\, (59\%)$
Height (*m*)	$1.70 \pm 0.12$
Weight (*kg*)	$69 \pm 11$
BMI ($\text{kg/m}^2$)	$24.7 \pm 2.7$
**Ethnicity**	
Caucasian	78
New Zealand $\hbox {M} {\overline{a}}\hbox {ori}$	1
Asian	1
African American	1
Unknown	2

*SD* standard deviation, *N* sample size.

**Figure 1:**
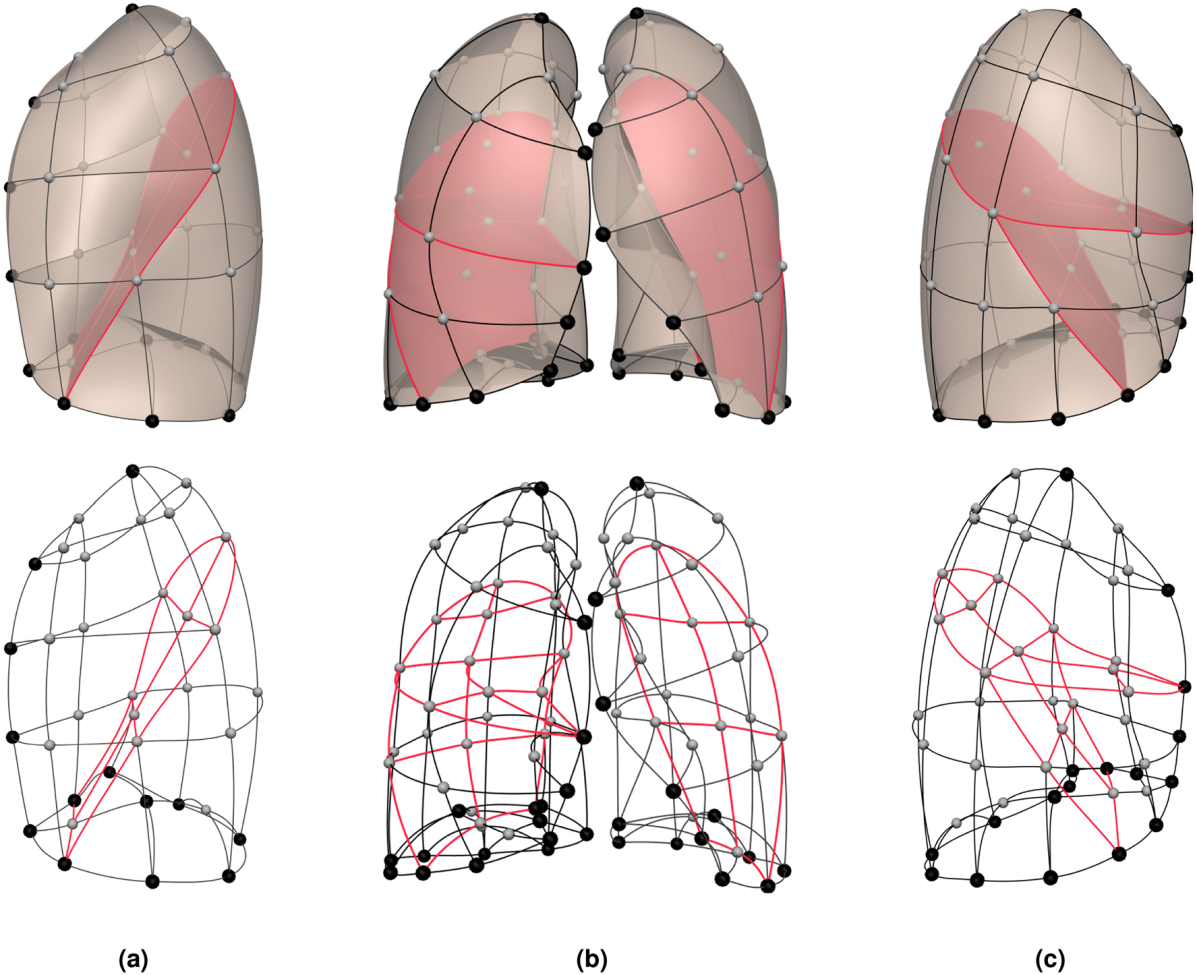
Finite element mesh topology used to construct subject-specific lung shapes. (**a**) Lateral view of the left lung, (**b**) front view of both lungs, and (**c**) lateral view of the right lung. Lobar fissures are also highlighted. The larger black spheres indicate the nodes used as anatomical landmarks, the smaller grey spheres indicate the nodes used as pseudo landmarks, and the lines indicate the interpolation edges that are used as the description of surface curvature—note that the fissure is shown in red colour. This approach to describe the shape ensures that the entire lung surface is taken into account in the statistical modeling calculations.

Volumetric imaging had slice spacing 0.5–0.7   $\hbox {mm}$ with reconstruction matrices of $768\times 768$ (> 50 years old) or $512 \times 512$ (< 50 years old). Scan parameters were 120 kV, 100 mAs, and pitch of 1.2. Lung and fissure surfaces were segmented using custom-written software^19^ with manual correction of fissures as required. To define the lung shape a high-order finite element (FE) mesh was geometry-fitted to the lung and fissure surfaces^20^ using the same mesh topology for all subjects (Fig. 1). The generic FE mesh had 35 nodes and 44 elements covering the left lung and left oblique fissure surfaces, and 50 nodes and 62 elements covering the right lung and right oblique and horizontal fissure surfaces. In order to provide anatomical and mathematical shape correspondence between subjects, anatomical landmarks were placed at the lung apices, dome of the diaphragm, the edge of the lung base, and the edge of anterior segments. Additional pseudo-landmarks were regularly-spaced between these points. The generic mesh was geometrically fitted to each subject’s segmented lung image using a linear least squares optimization^20^. Briefly, the sum of the Euclidean distances between each data point and its projection onto the nearest element was minimized during the fitting process. This distance is a function of the node location and shape parameter, i.e. its derivatives. The points are fitted by minimizing the energy function *T*(*u*) with respect to the global shape parameters:

1 $$\begin{aligned} T(u) = \sum \gamma \Vert z(\xi _{1},\xi _{2}) - z_{d}\Vert ^{2} + \int \limits _\varOmega g(u(\xi )) d\xi , \end{aligned}$$

where *u* is a vector of mesh parameters, $\gamma$ is a weight factor for each point, *z* is geometric position which can be given by local element coordinate $\xi$, $z_{d}$ is the spatial coordinates of the data point, and $\varOmega$ is the mesh domain. *g* is a second order weighted norm which is calculated from the derivatives of the geometry of each element^21^. This term is a smoothness constraint which measures the deformation of the surface, and was included to regularize the problem since data can sometimes be either insufficient or noisy. The root mean squared error for mesh fitting across the cohort was $5.2 \pm 2.3 \, \hbox {mm}$.

### Statistical shape analysis: SSA

We derived two statistical shape models (SSM) for the cohort using a principal component analysis (PCA). An SSM provides a mathematical description of the shape of an object and the ways that its shape varies. It captures the global shape of the object of interest instead of using fixed geometric measurements such as lengths and angles^22^. PCA is a mathematical technique that decomposes the shape of a generic object into its main components such that the weighted sum of these components can retrieve back the object’s shape. In our approach, the first model included lung size (using raw unscaled data; the ‘size-inclusive’ model) and the second model controlled for lung size (using scaled data; the ‘size-exclusive’ model). The mean shape and principal modes of shape variation were derived for the whole cohort for both the size-inclusive and size-exclusive models. The process of deriving the SSM involves three main steps: data alignment, data assembly, and data decomposition.

#### Data alignment

A key first step in deriving any SSM is to align the object shapes to a chosen reference coordinate. General Procrustes Alignment (GPA)^23^ was used to align all meshes to the same axis, hence removing rotation and translation bias; and in the size-exclusive model it was also used to remove scaling bias. This step is analogous to the rigid registration that removes any rigid body changes to the shape of an object. Without this step, the SSM would include rotation and translation as a component of shape variation, which is not desirable.

For the data alignment, let *S* be a vector containing the concatenation of all 3D lung coordinates, i.e. $S = [x_1, y_1, z_1 \ldots ]$, to the reference (mean) surface (i.e. $1/N \sum _i^N(x_1^i) \sum _i(y_1^i) \sum _i(z_1^i) \ldots$, where *i* iterates over *N* cases). We can now define the aligned shape vector ${\bar{S}}$ by:

2 $$\begin{aligned} {\bar{S}} = \alpha RS + T, \end{aligned}$$

where *R* and *T* are the unknown rotation matrix and translation vector, respectively, which align shape *S*, and $\alpha \in {\mathbb {R}}$ is the overall scaling factor.

#### Data assembly

We assemble information about lung shape (here represented as finite element nodes and curvature information at each node) from all of the subjects in the cohort. Each subject’s lung shape is considered to be a single observation. Let $S_{L}$ be one such observation column containing the shape parameters. In this case:

3 $$\begin{aligned} S_{L}^T = [{\bar{u}}_{1}, {\bar{u}}_{2}, {\bar{u}}_{3},\ldots , {\bar{u}}_{p-2}, {\bar{u}}_{p-1}, {\bar{u}}_{p}], \end{aligned}$$

where ($^{\textit{T}}$) denotes transpose, *u* is the node containing 12 DoFs (four for each direction: *c*, $\frac{\partial c}{\partial \xi _{1}}$,$\frac{\partial c}{\partial \xi _{2}}$, $\frac{\partial ^{2} c}{\partial \xi _{1} \partial \xi _{2}}$, where *c* = [*x*, *y*, and *z*]). These DoFs together describe the lung shape for one subject in the cohort. For a general surface in 3*D* space, these DoFs describe both the location and the surface curvature. Furthermore, *p* represents the number of nodes for both lungs and the overline ($^{\_}$) operator symbolizes the GPA to the reference lung model.

A new data matrix ${\mathbf {S}}$ is built by a concatenation of each lung observation $S_L$ to construct a lung training set. The full training set can be described as a set of *N*3*D* shapes, where *N* is the number of subject lung shapes. Each of these lung shapes is represented by a set of *n* landmarks, where *n* is the number of nodal shape parameters (i.e. 2700 for each subject, equalling 225 nodes times the number of nodal parameters of coordinates and derivatives for each node). By assembling the shape parameters this way, every lung shape has been converted from a 3D space to a single point in 3*nD* space. This 3*nD* space constitutes an ‘allowable shape domain’. ${\mathbf {S}}$ can be seen as a cloud of *N* points in the constructed 3*nD* space that lies within this domain^24^. Once such a space is constructed, any allowable shape will have to be a member of this domain. Conversely, any shape that is extracted from this domain will be an allowable shape.

#### Data decomposition

Decomposition aims to reduce the complexity of the data. Once the data has been assembled, the data matrix ${\mathbf {S}}$ can be decomposed into modes of shape variation using a number of different techniques. The current study employs a linear decomposition of ${\mathbf {S}}$ by PCA within the allowable shape domain. One of the benefits of PCA is to find a linear space of eigenvectors $m_{l}$ where $l = 1,\ldots , L$, with *L* being much smaller than the number of variables in the original rectangular Cartesian space (in this case $L \, \ll 2700 \times 83 = 224,100$). In statistical terminology, this technique is referred to as reducing the dimension of the *feature* space.

### Principal component analysis

PCA was performed by centering the data in ${\mathbf {S}}$ around the mean, i.e. ${\mathbf {S}} = \mathbf {S_0} - \bar{\mathbf {S_{0}}}$ where $\mathbf {S_{0}}$ is the original data matrix and $\bar{\mathbf {S_{0}}}$ is the mean of $\mathbf {S_{0}}$. Next the covariance matrix of ${\mathbf {S}}$ was built by ${\mathbf {C}} = {\mathbf {S}}{\mathbf {S}}^T$. Mean-centered data matrix ${\mathbf {S}}$ was then factorized using singular value decomposition (SVD) to yield:

4 $$\begin{aligned} {\mathbf {S}} = {\mathbf {U}}{\varvec{\varSigma }} \mathbf {V^T} , \end{aligned}$$

where ${\mathbf {U}}$ is an $m \times m$ unitary matrix of eigenvectors of ${\mathbf {S}}{\mathbf {S}}^T$, ${\varvec{\varSigma }}$ is an $m \times n$ rectangular diagonal matrix with non-negative square roots of the eigenvalues of ${\mathbf {S}}{\mathbf {S}}^T$, and ${\mathbf {V}}$ is an orthonormalized $n \times n$ unitary matrix containing eigenvectors of ${\mathbf {C}} = {\mathbf {S}}{\mathbf {S}}^T$. ${\mathbf {U}}$ is a rotational representation of the lung matrix ${\mathbf {S}}$^25^. Assuming that all dimensionality is preserved, the PCA transformation can be expressed by ${\mathbf {U}}$ using ${\mathbf {X}} = {\mathbf {U}}^T{\mathbf {S}}$. The elements in each column of ${\mathbf {U}}$, namely *u*, are the new variables transformed into the PCA-space ${\mathbf {X}}$. One column (i.e. $u_{l}$) represents a mode of variation or *principal components*, ordered by the singular values of ${\varvec{\varSigma }}$, namely $\sigma {_{l}}$. The singular values are proportional to the size of the variance corresponding to each eigenvector. This clearly shows that the variance explained by each component $u_{l}$ can be derived as $\sigma {_{l}} \equiv \sqrt{\lambda _{l}}$, where $\lambda _{l}$ are the eigenvalues of ${\mathbf {C}}$. The corresponding eigenvalues of ${\mathbf {C}}$ show how significant the components $\sigma$ are: a larger eigenvalue means a more significant eigenvector, hence a more significant mode of variation.

Shape modes are independent descriptors of shape and can be expressed as a percentage of the total shape variation in the population. Modes of variation are defined as perturbations about the mean and can be noted as ${\textit{m}}_{l}$ for each principal component $u_{l}$. In this case:

5 $$\begin{aligned} m_{l}(w) = {\bar{S}}_{0} + wu_{l} \equiv M_{l} , \end{aligned}$$

where *w* is a weight factor given to each mode of variation and $l = 1,\ldots ,L$. Variations in *w* within suitable limits (often between $\pm \,2-3\sigma$) allows for a direct visualization of the lung shape and shape changes. Each subject’s weight values for each mode of variation were calculated by projecting the subject onto the trained population’s PCs. The projection is performed by using the dot product of the PCA variance matrix and the subject’s shape vector:

6 $$\begin{aligned} s = (X_{i} - s_{mean}) \cdot {\mathbf {R}}, \end{aligned}$$

where *s* is the array of PC scores, $X_{i}$ is the desired subject dataset, $s_{mean}$ is the mean shape vector, and ${\mathbf {R}}$ is the population PCA variance matrix. The scores in the array *s* were then converted into relative standard deviation (S.D.) weight values.

### Mode selection and geometrical analysis

The number of shape modes obtained as a result of a PCA analysis is typically large. It is impractical and also not useful to study all of the shape modes. We employed the cumulative percentage of total variation as the selection criterion. The number of modes included in the analysis is then the smallest value of m for which this chosen percentage cut-off is exceeded. Modes are successively selected to have the largest possible variance. Let the variance of the *k*th mode be denoted as $l_k$. Then the sum of the variances of the modes is equal to the sum of the variances of the elements of *S* (our data; also note that *p* indicates those variables in *S*), which when expressed mathematically:

7 $$\begin{aligned} \sum \limits _{k=1}^p l_k = \sum \limits _{j=1}^p s_{jj}, \end{aligned}$$

As a result, the definition of ‘percentage of variation’ $v_m$ is:

8 $$\begin{aligned} v_m = 100 \times \frac{\sum \limits _{k=1}^m l_k}{\sum \limits _{j=1}^p s_{jj}} = 100 \times \frac{\sum \limits _{k=1}^m l_k}{\sum \limits _{k=1}^p l_k}, \end{aligned}$$

A sensible cut-off for $v_m$ often depends on the nature of a particular data. When *p* is very large (like in our case which is 224,100) we end up with an impractically large value of *m* for further analysis. For large *m*, geometrical interpretation of the modes become extremely difficult or even impossible^26^. It is also important to retain as many *m* as feasible to interpret and examine. As a result, a cut-off was chosen empirically by examining all of the modes and retaining those which showed the possibility of interpretation. For the significant modes that were chosen, a careful and detailed approach using 3*D* graphical visualization techniques was employed to derive information regarding the topological and geometrical changes in lung shape.

### Statistical analysis

Weightings for the first four shape modes for each model were tested for relationships with age, BMI, sex, and lung volume using ordinary least squares regression and Pearson correlation. Means of the 50 year old groups were compared using one-way ANOVA. For age, we used a standard two-sided independent t-test with unequal population variance (Welch’s *t*-test). A confidence level ($\alpha$) of 0.05 was considered statistically significant.

## Results

To confirm that the inclusion of data from different centers did not influence the imaged lung volume (and hence potentially the shape), the imaged full inspiratory supine lung volume as a proportion of plethysmographic (upright) assessed total lung capacity (TLC) was compared between subjects from the two centers (the $>50$ and $<50$ year old groups). There was no significant difference ($\hbox {p} = 0.15$) in the relative lung volume during imaging (ratios were $0.88 \pm 0.14$ and $0.93 \pm 0.08$ for the $>50$ y.o. and $<50$ y.o. group, respectively).

### Qualitative descriptions of modes of lung shape variation

**Figure 2:**
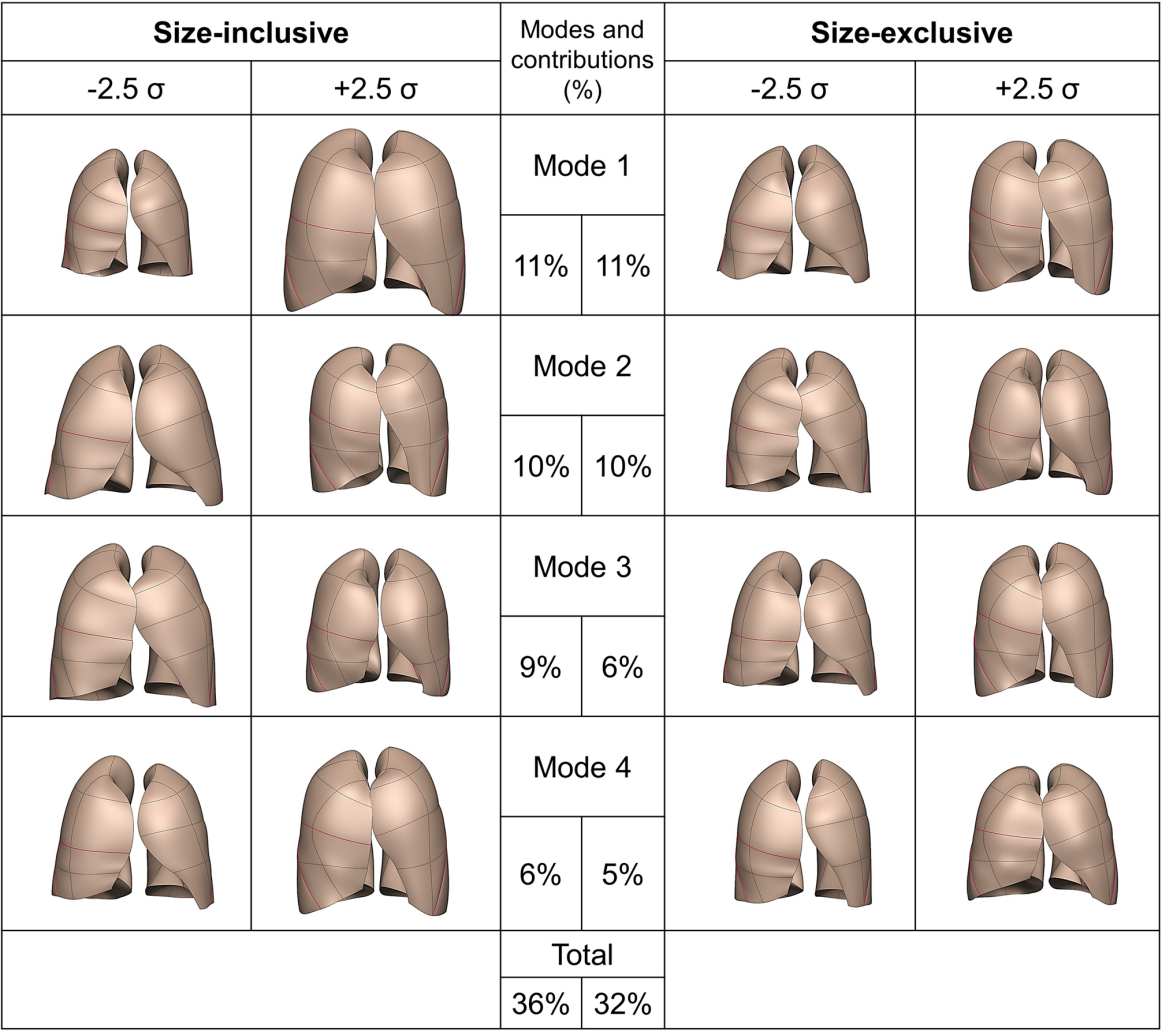
Lung shape at ± 2.5 SD from the mean for each of the first four principal modes, for the size-inclusive (left column) and size-exclusive (right column) statistical shape models. The left main panel illustrates shape modes 1 to 4 for the size-inclusive model, and the right main panel for the size-exclusive model. The middle column shows the relative contribution of each mode as a percentage of the total population shape variance.

Two separate shape models were derived to enable evaluation of the contribution of size (volume) to shape: the size-inclusive model and size-exclusive model. Lung shape for ± 2.5 standard deviations from the mean are illustrated in Fig. 2 for the first four principal modes, for the size-inclusive and -exclusive models. For both models, the first mode explained $11\%$ of the shape variation and the second explained $10\%$. Modes 3 and 4 explained $9\%$ ($6\%$) and $6\%$ ($5\%$), respectively for the size-inclusive (-exclusive) model; that is, the first four modes explained a total of $36\%$ ($32\%$) of the shape variation in the cohort.

In Fig. 2, negative weightings for mode 1 in the size-inclusive model (tending to be female) corresponded to smaller lungs with thinner anterior edges; positive weightings corresponded to larger lungs (tending to be male) and thicker anterior edges that almost touched each other, with locally dilated regions. In contrast, in the size-exclusive model the first mode corresponded mainly to the antero-posterior diameter of the lung with a mediolateral narrowing towards the apices for negative weightings, and relatively constant mediolateral dimension from base to apex for positive weightings. In addition, this mode in the size-exclusive model showed a large shift in fissure locations in both the right and left lungs with mode weighting: the horizontal fissure descended towards the base as weighting became more negative, and the oblique fissures retracted posteriorly. The second principal mode for the size-inclusive model showed a very similar pattern of variation to the first principal mode for the size-exclusive model, explaining $~10\%$ of the cohort’s variation in shape.

For the second shape mode of the size-exclusive model, the shape variability involved an inwards rotation of the anterior aspect of each lung about the cranio-caudal axis, such that the upper anterior region of each lung moved closer to each other for negative weightings. The third mode for the size-inclusive model showed a very similar pattern of variation to this. The size-exclusive mode 3 and size-inclusive mode 4 presented some degree of aspect ratio change (that is, the ratio of the width to the height of lung). The size-exclusive mode 4 presented a more aggressive aspect ratio change with a more inflated and ‘barrel-shaped’ lung for positive weightings, and a less inflated and elongated lung for negative weightings. This shape mode also showed some rotation about the cranio-caudal axis, with the anterior of the lungs rotating towards each other for positive weightings.

### Associations between lung shape, sex, and size

All shape modes were examined for associations with age, sex and lung size. Only the modes that have significant relationships with physiological or anthropometric data are illustrated, except for sex differences.

**Figure 3:**
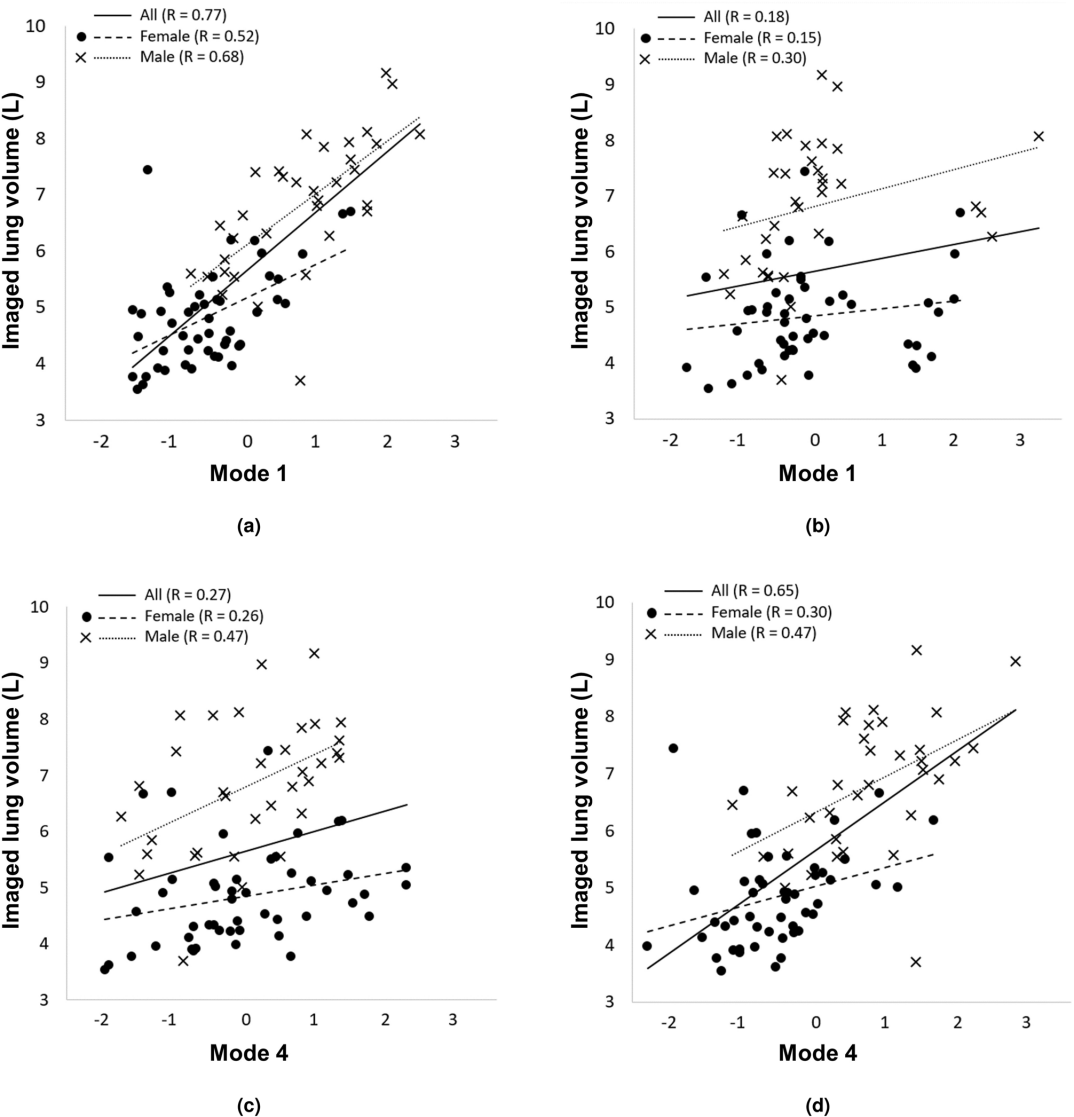
The relationship between total imaged lung volume and principal shape modes 1 and 4 for the (**a**,**c**) size-inclusive and (**b**,**d**) -exclusive models. Data for females (filled circles) and males (crosses) are indicated separately. Linear regressions to the data are shown for the whole cohort (solid lines) and separately for females (heavy dashed lines) and males (light dotted lines). The size-inclusive mode 1 and size-exclusive mode 4 are strongly associated with volume ($\hbox {R}=0.77$, $\hbox {p}<0.001$, and $\hbox {R}=0.65$, $\hbox {p}<0.001$, respectively). Significant relationships are not apparent for the other two model modes (R=0.18, p=0.10 for size-exclusive mode 1, and R=0.27, p=0.08 for size-inclusive mode 4).

As shown in Fig. 3a, when volume was not controlled for the first principal shape mode was strongly associated with lung size ($\hbox {R} = 0.77$, $\hbox {p} < 0.001$), with the female data tending towards negative, and male data positive, weightings. The relationship for the whole cohort was stronger than for females ($\hbox {R} = 0.52$, $\hbox {p} < 0.001$) or males ($\hbox {R} = 0.68$, $\hbox {p} < 0.001$) considered separately. In contrast, mode 1 was not associated with lung size in the size-exclusive model ($\hbox {R} = 0.18$, p = 0.10), but mode 4 was ($\hbox {R} = 0.65$, $\hbox {p} < 0.001$) when considering the entire cohort. While Fig. 2 suggests qualitatively that size could be a factor in modes 2–4 of the size-inclusive model, this is not borne out statistically: size-exclusive mode 4 (Fig. 3d) is the only other shape mode that shows a significant association with lung volume ($\hbox {R} = 0.65$, $\hbox {p} < 0.001$). Similar to the distribution of data for mode 1 of the size-inclusive model (Fig. 3a), mode 4 in Fig. 3d shows females tending to negative weightings and males to positive weightings.

**Figure 4:**
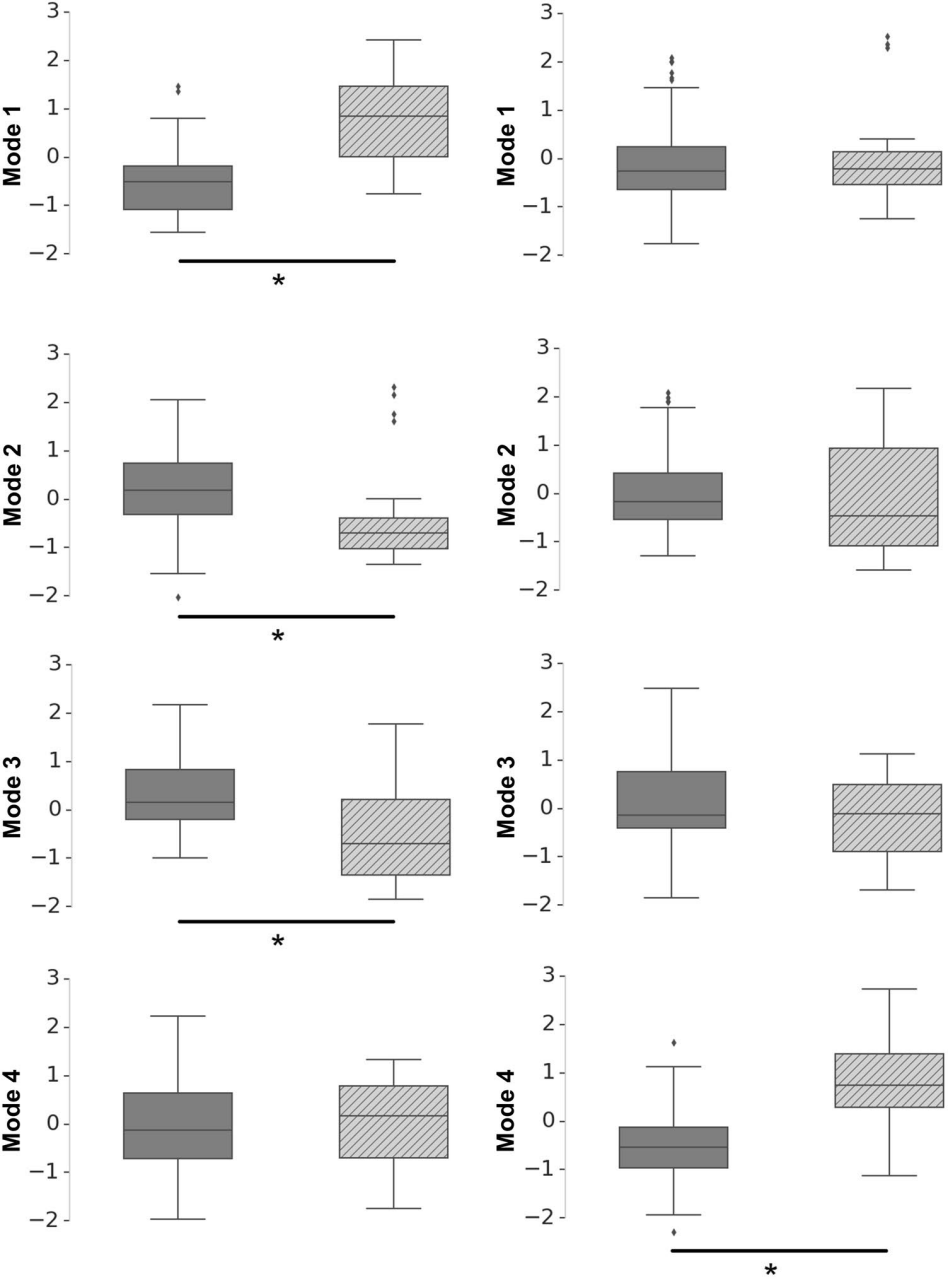
Distribution of mode weightings for the size-inclusive (left column) and size-exclusive (right column) models, for females (grey) and males (cross-hatched). The shape modes 1–4 are shown from top to bottom. Statistical significance is indicated using ‘*’. Modes 1–3 in the size-inclusive model and mode 4 of the size-exclusive model have statistically significant differences between males and females.

Figure 4 and Table 2 summarize the distributions of shape mode weightings when grouped by sex. The first three shape modes for the size-inclusive model are significantly different ($\hbox {p} < 0.001$ for mode 1, p = 0.002 for mode 2, and $\hbox {p} < 0.001$ for mode 3) between males and females, but the fourth mode is not. In contrast, the size-exclusive model modes 1 to 3 have no sex differences, whereas the male/female difference in mode 4 is significant ($\hbox {p} < 0.001$). For the size-inclusive model, males and females tend towards negative and positive weightings for mode 1, respectively.

**Table 2:** Mean (± standard deviation) of the first four principal shape mode weightings for males and females for a size-inclusive and a size-exclusive statistical shape model.

	Size-inclusive model			Size-exclusive model		
	Female (mean ± SD)	Male (mean ± SD)	p-value	Female (mean ± SD)	Male (mean ± SD)	p-value
Mode 1	− 0.50 ± 0.72	0.77 ± 0.84	< 0.001*	− 0.01 ± 0.98	0.04 ± 1.03	0.83
Mode 2	0.28 ± 0.96	− 0.40 ± 0.94	0.002*	1.31 ± 0.92	− 0.16 ± 1.10	0.20
Mode 3	0.38 ± 0.83	− 0.54 ± 0.98	< 0.001*	0.13 ± 1.10	− 0.20 ± 0.87	0.14
Mode 4	− 0.03 ± 1.06	0.03 ± 0.91	0.80	− 0.52 ± 0.75	0.74 ± 0.84	< 0.001*

Corresponding p-values from an independent t-test for females and males are shown, with $\hbox {p} < 0.05$ (indicated by $*$) considered statistically significant.

### Associations between lung shape and age

**Figure 5:**
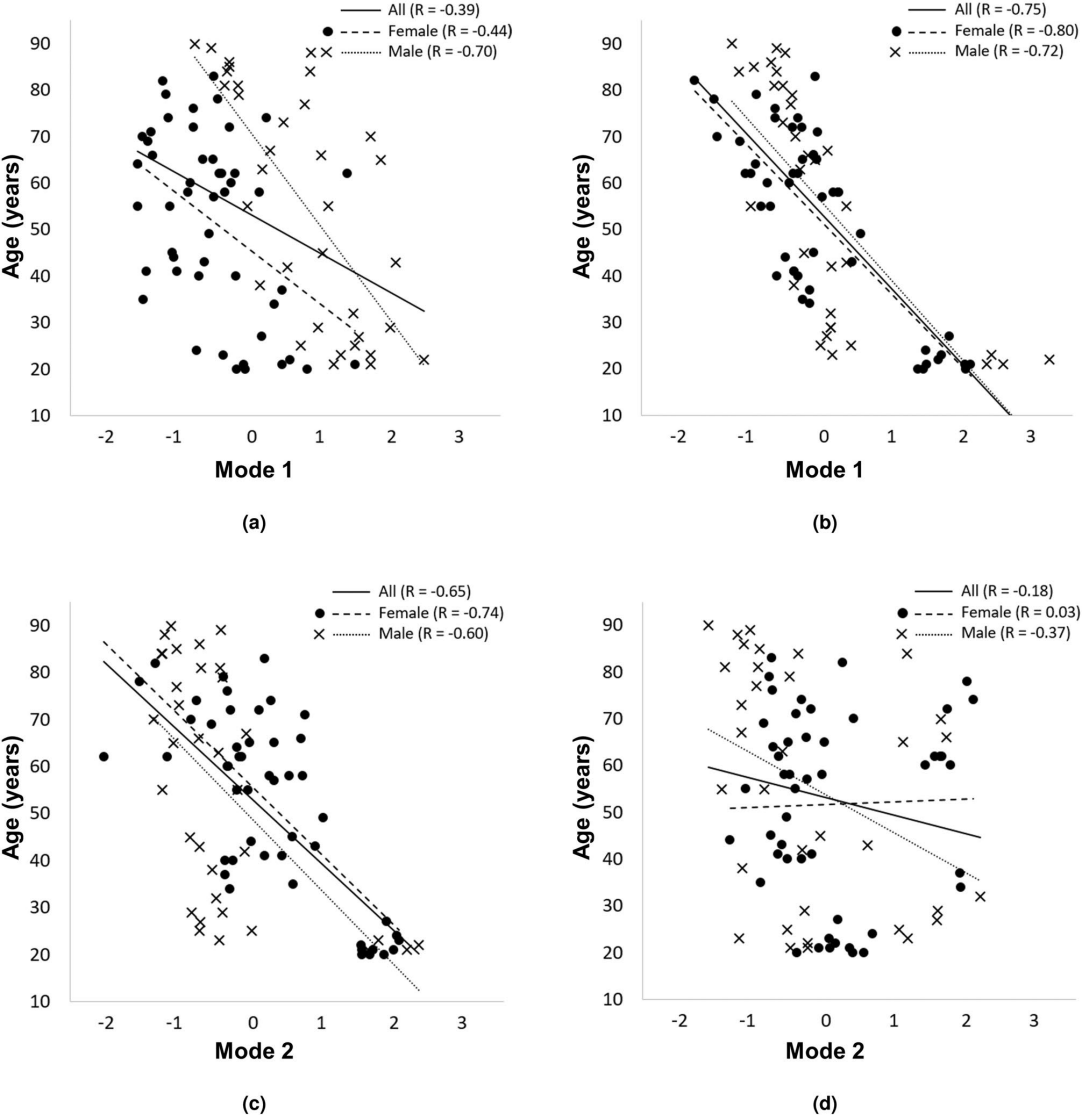
The relationship between age and shape modes 1 and 2 for the (**a**,**c**) size-inclusive and (**b**,**d**) size-exclusive models. Data for females (filled circles) and males (crosses) are indicated separately. Linear regressions to the data are shown for the whole cohort (solid lines) and separately for females (heavy dashed lines) and males (light dotted lines). Qualitatively, size-inclusive mode 1 captures the lung size, size-inclusive mode 2 and size-exclusive mode 1 capture a change in the antero-posterior dimension when moving from base to apex as well as a large shift in fissure location, and size-exclusive mode 2 captures rotation about the medio-lateral axis. Age is strongly associated with mode 1 in the size-exclusive model ($\hbox {R}= {-}\,0.75$, $\hbox {p}<0.001$) and with mode 2 in the size-inclusive model ($\hbox {R}={-}\,0.65$, $\hbox {p}<0.001$). Age is moderately associated with mode 1 in the size-inclusive model (R = 0.39, $\hbox {p}\,<\,0.001$).

Age is strongly associated with shape for both the size-inclusive and -exclusive models in Fig. 5. Strong relationships with age are evident for size-exclusive mode 1 (Fig. 5b, $\hbox {R} = -\,0.75$, $\hbox {p} < 0.001$) and size-inclusive mode 2 (Fig. 5c, $\hbox {R} = -\,0.65$, $\hbox {p} < 0.001$). For both modes, the relationships for separate sexes are not different from that for the whole cohort. Age and size-inclusive mode 1 shows moderate association for the whole cohort (Fig. 5a, $\hbox {R} = -\,0.39$, $\hbox {p} < 0.001$), however separation by sex reveals a strong relationship for males with age ($\hbox {R} = -\,0.70$, $\hbox {p} < 0.001$), and a weaker (than male) but important association for females ($\hbox {R} = -\,0.44$, $\hbox {p} = 0.002$).

### Associations between lung shape and BMI

**Figure 6:**
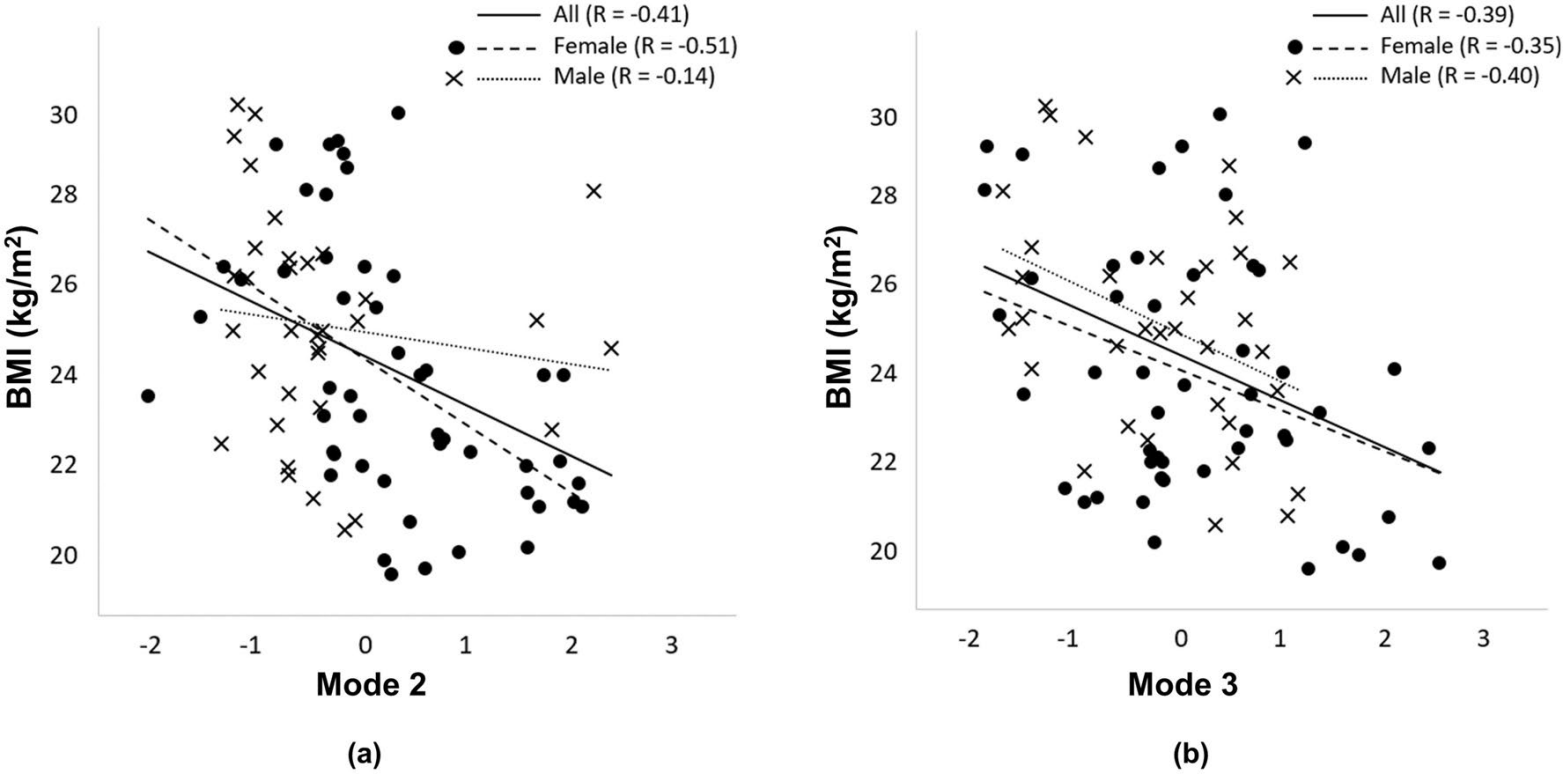
The relationship between (**a**) BMI and shape mode 2 for the size-inclusive model and (**b**) shape mode 3 for the size-exclusive model. Data for females (filled circles) and males (crosses) are indicated separately. Linear regressions to the data are shown for the whole cohort (solid lines) and separately for females (heavy dashed lines) and males (light dotted lines). Both modes for the respective models have moderately strong and significant relationships with BMI when considering the entire cohort. The relationship for males in the size-inclusive model is not significant ($p=0.43$) when considered separately.

The shape modes that appear to be associated with BMI are shown in Fig. 6. Size-inclusive mode 2 has a significant association with BMI ($\hbox {R} = -\,0.41$, $\hbox {p} < 0.001$). Separation by sex reveals a similar relationship for females ($\hbox {R} = -\,0.51$, $\hbox {p} < 0.001$) but no relationship for males ($\hbox {R} = -\,0.14$, $\hbox {p} = 0.43$) with BMI. For size-exclusive mode 3, the individual relationships for females ($\hbox {R} = -\,0.35$, $\hbox {p} = 0.02$) and males ($\hbox {R} = -\,0.40$, $\hbox {p} = 0.02$) with BMI are similar to that for the whole cohort ($\hbox {R} = -\,0.39$, $\hbox {p} = 0.01$).

## Discussion

Older age and chronic disease are both known to affect chest wall shape, lung dimensions, and lung function. Lung shape therefore potentially provides a prognostic marker of lung health or accelerated aging, but this has not previously been explored. To distinguish between normal and abnormal lung shape, a quantitative description of normal shape and its variability is first required. Our study provides a quantitative statistical shape description of the lung and its lobes in a cohort of 83 never-smoking healthy subjects aged 20–93 years. The statistical model shows a strong relationship between shape and age, and an association between shape and BMI. Male and female shape only differed when lung size was not controlled for in the model, which differs from previous studies of sex differences in lung shape^8^.

Rib cage geometry has been studied in detail by a number of authors, using quantitative methods to describe its normal shape and revealing relationships with age, BMI, and sex^1,10^. However, the rib cage only describes one component of lung shape: the diaphragm forms the additional boundary of the lung ‘container’, and the pulmonary fissures provide important information about lobar shape. Some other direct analyses of lung shape have relied upon measurements of linear dimensions or cross-sectional area at select locations^11–13^, but this cannot account for detailed features such as surface curvature or the location of the fissures. We used a PCA to derive statistical shape models for healthy, never-smoking, and radiologically normal subjects. The shape models represent the average shape of this cohort and its principal modes of shape variation ranked in order of the proportion of variation in shape that they explain. Any subject in the study cohort can be modeled as the mean shape plus a weighted sum of principal components, providing a compact quantitative description of individual subject shape. A further advantage of this approach is that it is straightforward to test whether a subject that was not in the training cohort can be considered to have the same shape as the cohort.

Average lung size is different between males and females, therefore we derived two shape models that allowed us to assess the contribution of lung size (volume) to the quantitative description of shape and therefore the appearance of sex differences. We expect that mode ($i + 1$) in the size-inclusive (not volume controlled) model captures similar shape information to mode (*i*) in the shape-exclusive (volume controlled) model. This is supported by the similarity of shape for modes ($i + 1$) to modes (*i*) (size-inclusive and -exclusive, respectively) in Fig. 2, and the corresponding data in Fig. 4. However, the size-inclusive model—by its nature—includes size variation as a shape feature in each of the first four principal shape modes (Fig. 2).

Our shape models show strong relationships between age and lung shape (summarized in Fig. 5), consistent with relationships between rib morphology and age^10^. The strongest relationship was with the first principal shape mode in the size-exclusive model. There were no sex differences in the relationship for this model/mode. Similar relationships with both age and sex differences were observed for mode 2 of the size-inclusive model, albeit with slightly weaker relationships that presumably reflect some contribution of lung size. Interestingly, when volume is not controlled for there is a much stronger relationship between the first principal shape mode and age for males compared with the whole cohort or females. The reason for these differences is not clear.

A more ‘pyramidal’ lung shape was observed with advancing age: that is, a smaller apical dimension compared with the diaphragmatic region in older subjects (Fig. 2). This was the same for both males and females (results not shown). These findings are consistent with previous studies that have found age-related alterations in thoracic shape, often as the consequence of deformity of the vertebral bodies leading to kyphosis^27^. Thoracic kyphosis leads to an increase in the anterio-posterior diameter of the chest which, in turn, results in an increased diameter of the lungs and the subsequent ‘pyramidal’ lung shape. In addition to differences in the exterior shape of the lung, a large displacement of the fissures with age was seen in both lungs (Fig. 2). The horizontal fissure descended towards the lung base in proportion to age, and the oblique fissures retracted posteriorly. The age-associated shape differences that we observed were therefore not just associated with chest wall remodeling, but also with a change in the configuration of the fissures.

Previous studies have reported that normal age-related changes to lung structure become most obvious from the beginning of the third decade of life^28^, and these changes in structure are associated with loss of static elastic recoil pressure of the lung^29,30^. We note that the large majority of the subjects aged greater than 30 years in our study had negative weightings for the size-exclusive mode 1 (i.e. in the direction of ‘older’ age in Fig. 5c) and almost every subject aged less than 30 years had positive weightings. That is, for the healthy cohort considered here, the average shape (zero SD weighting) was at about 30 years. This compares with an average cohort age of $53 \pm 22$ years. It is therefore likely that the change in shape—and particularly the fissure displacement—with age that we observe in this cohort reflects lung tissue micro-structural and elasticity changes, and their force balance with the chest wall. For example, the loss of elastic recoil with age leads to airway closure and increase in RV. This implies that at full-inspiration, the dependent airways will be narrower with resultant changes in airways resistance and compliance impacting on lung volume, and this indirectly acting on the position of the fissures.

In our model, males and females differ in their lung shape for the first three principal shape modes only when size (i.e. lung volume) is included in the analysis, as illustrated in Fig. 4. Mode 4 of the size-exclusive model shows apparent sex differences, however this mode is strongly associated with lung volume. In our dataset, full-inspiratory imaged lung volume is significantly larger ($\hbox {p} < 0.001$) in males ($6.81 \pm 1.16 \, \hbox {L}$) than females ($4.84 \pm 0.85 \, \hbox {L}$). The relationship between lung volume and mode 4 in Fig. 3d) shows overlap between male and female lung size, with smaller male lungs tending towards negative weightings along with most female lungs, and larger female lungs tending towards positive weightings along with most of the males. That is, the mode’s apparent association with sex is because of the difference in average volume of male and female lungs. Our general lack of sex differences contrasts with studies of rib cage morphology, which describe a rounder (less elliptical) lung cross-section in females^9,12^. It also contrasts with a recent study that used similar methods to quantify shape differences between males and females^8^. This study found a $7\%$ sex difference in shape in healthy subjects (21 females and 19 males) aged $51.9 \pm 1.2$ years, and described males as having a ‘pyramidal’ geometry and females having a ‘prismatic’ shape. Interestingly, this is qualitatively similar to the extremes of the size-exclusive first principal shape mode in our study, which we found was strongly associated with age. A key difference between our study and others is that we explicitly included the pulmonary fissures. As previously explained, we found that the fissure location changes with age and this change is the same for males and females. It is therefore likely that in our cohort and model the fissure location is a more dominant shape feature than the external lung shape (which could have more-apparent sex differences). A further point is that most previous studies do not normalize for lung volume. This is not important when using ratios of linear dimensions (e.g. anterio-posterior diameter/lateral diameter) but is important for 3D analyses such as PCA. This is apparent in the size-inclusive model (Fig. 2), where size is the dominant feature of the first shape mode (Fig. 3) and contributes to significant sex differences in Table 2 and Fig. 4. One methodological difference is that our study used far fewer ’landmark’ points than other similar studies (e.g. in comparison to the 12 anatomical and 403 ‘semi-landmarks’ of Torres-Tamayo et al.^8^). That is, our approach has far fewer points at which the surface location is sampled. However, our high-order FE mesh description includes surface curvature and smoothness such that additional intermediate points (between landmarks) could be interpolated as a function of the geometric information at the landmark nodes. That is, both approaches contain similar information, but stored in different forms.

BMI is associated with lung shape in Fig. 6, which is consistent with previous studies^13^. However, the relationship with BMI is only apparent in the third principal shape mode (size-exclusive model). That is, the contribution to shape is quantifiable but qualitatively subtle. Mode 2 of the size-inclusive model and mode 3 of the size-exclusive model have similar strength of relationship with BMI when considering the entire cohort. However, separation by sex shows no relationship for males with BMI when size is not controlled for in the model. The impact of BMI on lung and lobe shape appears to be similar between males and females when we control for volume. Our cohort did not include subjects with $\hbox {BMI} >30 \, \hbox {kg/m}^2$, hence we had a relatively narrow BMI range in the study. It is possible that the inclusion of subjects with higher BMI would reveal a stronger association with lung shape. Conversely, the inclusion of subjects with high BMI could decrease the strength of the relationship due to air trapping (when imaged supine) restricting lung shape.

One limitation of this study is that the cohort was not ethnically uniform. It is possible that genetic differences between different ethnic groups (e.g. European-American or -New Zealander, Asian-American) is associated with lung shape. We repeated our analysis after excluding five subjects who were identified as of non-European (’caucasian’) descent. A two-tailed non-parametric t-test between the results of our study and the new modes revealed no significant differences ($\alpha > 0.05$). It is possible that an analysis using a far larger cohort could reveal differences due to ethnicity, however the small ethnic diversity in our study does not appear to have influenced our results.

The first four principal shape modes only explained 32–36% of the shape variability in this cohort. The relevance of other modes was examined (not shown here) but no associations with physiologic or anthropometric data were found. It is possible that the addition of more subjects, or examination of a separate cohort, could yield different principal shape modes that explain more shape variation in the first few modes. A leave-one-out analysis of the current cohort shows no significant impact on the shape modes.

Our study only quantified shape in the supine lung at full-inspiration. A change to the upright posture is likely to modify lung shape, but we do not have data to determine how variable this is or if there is an association with age, sex, or BMI. It is possible that the stiffer chest wall in older subjects limits the range of ‘container’ shape change (except at the diaphragm) more than in young subjects with a compliant chest wall, which would imply a stronger relationship between age and upright shape. Differences in tissue elasticity with age might also be more apparent in the upright lung (with gravity acting over a greater height than when supine), hence exaggerating differences in fissure location.

We only examined static lung shape from imaging acquired at full-inspiration because this is the volume at which clinical imaging is most frequently acquired. Other studies have found sex differences in the change in shape of the ‘container’ of chest wall and diaphragm from end-expiration to full-inspiration^8^. It is not clear how the presence of fissure shape would influence sex differences for a similar analysis using our model.

This study is a first step towards understanding age-related changes of lung shape that presumably impact on lung function, and establishing lung shape as a potential biomarker of healthy aging. The shape of the lung is straightforward to extract from clinical imaging, such as CT or even from non-ionizing sources such as magnetic resonance imaging. Therefore, if shape is shown to be associated with age, it could provide a straightforward means for staging or stratification of lung disease. We have shown very clear changes in lung shape with age that are not simply the result of changes in the rib-cage geometry. These effects of age almost certainly reflect a complex interplay between changes in lung parenchyma and chest wall compliance. The physiological importance of these relative changes remains to be explored.
